# High-fat diet-induced obesity impairs endothelium-dependent relaxation in rabbits: association with MLCK upregulation and partial *ex vivo* improvement by ML-7

**DOI:** 10.3389/fcvm.2026.1797300

**Published:** 2026-05-11

**Authors:** Jiao Li, Jiantao Zhou, Qingxu Ha, Junwei Gu, Hui Yuan, Juan Cheng, Xiaojun Zha, Jingjing Teng, Liang Li, Junli Ding

**Affiliations:** 1 Department of Pediatrics, First Affiliated Hospital of Anhui Medical University, Hefei, China; 2 Anhui Center for Disease Control and Prevention, Public Health Research Institute of Anhui Province, Hefei, China; 3 Department of Physiology, School of Basic Medical Sciences, Anhui Medical University, Hefei, China; 4 Department of Biochemistry & Molecular Biology, School of Basic Medicine, Anhui Medical University, Hefei, China; 5 Department of Ultrasound, First Affiliated Hospital of Anhui Medical University, Hefei, China; 6 China National Clinical Research Center of Respiratory Diseases, Capital Medical University, Beijing, China

**Keywords:** dyslipidemia, endothelial dysfunction, ML-7, MLCK, obesity, PRAT

## Abstract

**Objective:**

Obesity is an independent risk factor for cardiovascular diseases and elevated mortality, yet the underlying mechanisms remain incompletely understood. Impaired endothelium-dependent relaxation is an early manifestation of vascular injury. This study investigated the impact of high-fat diet (HFD)-induced obesity on endothelium-dependent relaxation function and explored the potential role of myosin light chain kinase (MLCK) signaling in a rabbit model.

**Methods:**

Forty-five male New Zealand White rabbits were randomized into control (normal diet), HFD, and ML-7 (HFD plus the MLCK inhibitor ML-7, 1 mg/kg/day) groups (*n* = 15/group). After 8 weeks, *in vivo* endothelial function was assessed by flow-mediated dilation (FMD) of the iliac artery. Serum lipids and fasting glucose levels were measured. *Ex vivo* endothelium-dependent and endothelium-independent relaxations were evaluated in aortic rings using cumulative concentrations of acetylcholine (ACh, 0.001–10 μmol/L) and sodium nitroprusside (SNP). Aortic protein expression levels of MLCK, endothelial nitric oxide synthase (eNOS), and phosphorylated myosin light chain (p-MLC) were analyzed by Western blotting.

**Results:**

HFD-fed rabbits developed obesity (body weight +27.85% vs. control), dyslipidemia (elevated TC, LDL-C, and ox-LDL; all *P* < 0.001), hyperglycemia (*P* < 0.05), and significant endothelial dysfunction, characterized by impaired FMD (*P* < 0.05) and reduced ACh-induced relaxation of aortic rings. This was accompanied by increased aortic MLCK expression (*P* < 0.001) and a higher p-MLC/MLC ratio (*P* < 0.001), whereas eNOS expression and arterial NO levels remained unchanged. ML-7 treatment significantly reduced MLCK expression and MLC phosphorylation. ML-7 partially improved ACh-induced relaxation in aortic rings at higher concentrations (1 and 10 μmol/L, *P* < 0.05 vs. HFD) but failed to enhance FMD in the iliac artery *in vivo*. SNP-induced, endothelium-independent relaxation was similar across all groups.

**Conclusions:**

Obesity-induced endothelial dysfunction is associated with visceral fat accumulation, dyslipidemia, and MLCK upregulation. MLCK inhibition with ML-7 partially restored endothelium-dependent relaxation *ex vivo* in a concentration-dependent manner but did not translate to improved FMD *in vivo*. This dissociation highlights the complex nature of vascular dysfunction and suggests that noninvasive FMD may not capture modest, pathway-specific improvements, warranting caution in its use as a sole surrogate for endothelial health in preclinical studies.

## 1 Introduction

Obesity is a chronic disease that severely impacts physical and mental health, reduces quality of life (1), and imposes significant medical costs (2). Its prevalence has been continuously increasing across all age groups in China (3), with the prevalence of obesity among adults increasing from 3.1% in 2004 to 16.4% during 2015–2019 (4). Asian children, particularly Chinese children, exhibit higher body fat percentages and central obesity (5, 6), which are closely associated with an increased risk of atherosclerosis (AS) (7). The 2021 Global Burden of Disease Study revealed that China ranks first in terms of the absolute burden of obesity (8). These statistics highlight the obesity epidemic and its heavy burden.

Obesity exacerbates inflammation and oxidative stress, leading to severe complications such as cardiovascular diseases (CVDs) (9–13). Atherosclerosis, the pathological basis of most CVDs, often begins in childhood through a long and complex process; childhood obesity accelerates AS progression, leading to early-onset CVD (14, 15). Therefore, detecting its initial stage—endothelial dysfunction (ED)—is crucial (16). ED includes impaired endothelium-dependent relaxation, compromised barrier function, and vascular inflammation. Noninvasive flow-mediated dilation (FMD) has potential for predicting endothelial function but requires further validation (17).

ED is influenced by various factors, including genetics, hypertension, hyperlipidemia, diabetes, obesity, aging, smoking, and physical inactivity (18), but the underlying mechanisms remain to be fully elucidated. Our previous research revealed that upregulation of myosin light chain kinase (MLCK) expression and subsequent phosphorylation of myosin light chain (MLC) exacerbated ED associated with AS; notably, the MLCK inhibitor ML-7 effectively alleviated this dysfunction (19). Additionally, ED induced by obesity is closely linked to reduced nitric oxide (NO) bioavailability and endothelial nitric oxide synthase (eNOS) uncoupling (20–22). In obese mice, eNOS uncoupling leads to decreased NO production, contributing to aortic dysfunction (22).

This study aimed to investigate changes in vascular endothelium-dependent relaxation [acetylcholine (ACh)-induced aortic ring relaxation] in high-fat diet (HFD)-induced obese rabbits and their relationships with risk factors such as blood lipids and glucose, with a particular focus on associations with MLCK expression/activity, eNOS expression/activity, and NO levels. These investigations help elucidate the mechanisms underlying obesity-induced endothelial dysfunction and compare the findings from noninvasive FMD with those from *ex vivo* aortic ring testing, in order to better understand the relationship between these two assessment modalities.

## 2 Materials and methods

### 2.1 Animal experimental procedures

After a 2-week acclimatization period, 45 male New Zealand White rabbits [3 months old, 2.7 (2.5, 2.8) kg; Laifu Animals Breeding Center, Nanjing, China; SCXK(Su)2019-0005] were randomly allocated into three groups (*n* = 15/group) using a computer-generated random number table and housed under identical conditions for 8 weeks. The HFD group was fed a HFD (0.5% cholesterol, 5% lard, 5% soybean oil, and 89.5% standard chow) plus ultrapure water by oral gavage daily. The ML-7 group received the same HFD plus daily oral ML-7 (1 mg/kg; DC Chemicals, China; DC2052) in ultrapure water. The control group was fed standard chow plus ultrapure water by oral gavage daily. Oral gavage was performed daily between 9:00 and 11:00 AM using a silicone catheter; the procedure was uniformly applied to all groups to control for stress. After 8 weeks, all the animals were euthanized for tissue collection. All procedures were approved by the Ethics Committee of the Anhui Provincial Center for Disease Control and Prevention (Approval No. 2023006). The primary outcome was endothelium-dependent relaxation, which was assessed by ACh-induced aortic ring relaxation. Secondary outcomes included FMD, Oil Red O (ORO), hematoxylin and eosin (HE) staining, protein expression (MLCK, p-MLC, MLC, and eNOS), arterial NO content, serum parameters [lipid profile, fasting glucose (Glu)], and perirenal adipose tissue (PRAT).

### 2.2 Terminal procedures: ultrasound assessment, anesthesia, and euthanasia

Immediately after the noninvasive ultrasound assessment of flow-mediated dilation in the left external iliac artery (performed as described in Section 2.9), anesthesia was induced. A slow intravenous injection of a 3% pentobarbital sodium solution (30 mg/kg) was administered via the right marginal ear vein. A surgical plane of anesthesia was confirmed by the loss of corneal and toe-pinch reflexes. With the animal under stable, deep anesthesia, the hair on the neck was removed using a sterilized electric clipper, and the surgical site was disinfected again. A longitudinal incision approximately 6 cm in length was made along the median line of the neck. Subcutaneous fat and superficial fascia were bluntly dissected layer by layer using blunt-tipped hemostatic forceps to expose the platysma and sternocleidomastoid muscles. The muscle layers were separated along the direction of the muscle fibers using microsurgical scissors to expose the trachea and both common carotid arteries (care was taken to preserve the integrity of the vascular sheath). Whole blood was then slowly collected into a coagulation-promoting tube via puncture of the right common carotid artery.

While the animal remained under stable, deep anesthesia, euthanasia was subsequently performed by rapid intravenous injection of an overdose of pentobarbital sodium (150 mg/kg) via the same venous access. Death was confirmed by the permanent cessation of respiration and heartbeat and by pupils that were fixed and dilated with no response to strong light.

### 2.3 Tissue harvesting and processing

After euthanasia, the thoracic aorta was rapidly isolated through thoracotomy, placed in 4 °C Krebs solution (maintained on ice), and continuously aerated with a gas mixture of 95% O₂ and 5% CO₂. Adipose and connective tissues surrounding the aorta were carefully removed using ophthalmic instruments. A 6–8 mm segment of the aortic ring was excised for *ex vivo* vascular ring experiments, while the remaining tissue was preserved for subsequent analyses, including ORO staining and paraffin sectioning. PRAT situated between the renal capsule and renal fascia was collected and weighed (23).

### 2.4 Lipid profile and Glu measurements

Serum was collected from the auricular veins of New Zealand white rabbits. After the samples were allowed to clot for 2 h at room temperature, they were centrifuged (3,500 × g, 15 min, 4 °C) to isolate the serum. The supernatant was aliquoted into sterile tubes, wrapped in aluminum foil to protect it from light, and stored at −80 °C to avoid freeze‒thaw degradation. Glu and lipid profiles, including total cholesterol (TC), triglycerides (TG), low-density lipoprotein cholesterol (LDL-C), high-density lipoprotein cholesterol (HDL-C), oxidized low-density lipoprotein (ox-LDL), and very-low-density lipoprotein (VLDL), were quantified using commercial ELISA kits. The kits for Glu (JL-T1253), TC (JL-T1371), TG (JL-T0853), LDL-C (JL-T0807), HDL-C (JL17060), and VLDL (JL-33888) were obtained from Shanghai Jianglai Biological Technology Co., Ltd; the ox-LDL kit (JM-09021R1) was obtained from Jiangsu Jingmei Biological Technology Co., Ltd.

### 2.5 ORO staining

Aortic tissue was dissected and stained with ORO solution (prepared by dissolving 0.25 g of ORO in 50 mL of 60% isopropanol and diluting it with distilled water at a 3:2 ratio before staining) for 20 min. After staining, the tissue was washed with 60% isopropanol to remove unbound dye. The staining results were qualitatively analyzed by imaging to assess the distribution of fat infiltration in the aorta.

### 2.6 HE staining

Thoracic aortic specimens were trimmed into 5 mm thick sections, fixed overnight in 4% formaldehyde, dehydrated through an ethanol gradient, and embedded in paraffin. The sections were subjected to HE staining and observed under an optical microscope (Leica, Wetzlar, Germany) to examine the arterial wall structure.

### 2.7 Western blotting (WB)

Aortic tissue was subsequently washed three times with Tris-buffered saline and lysed using precooled radioimmunoprecipitation assay buffer, followed by centrifugation at 18,800 × g for 30 min at 4 °C. The total protein concentration was determined using a commercial assay kit (Elabscience, E-BC-K318-M), and a standard curve was constructed. Equal amounts of protein samples were separated by 10% sodium dodecyl sulfate–polyacrylamide gel electrophoresis and transferred to polyvinylidene fluoride membranes, which were then blocked with 5% nonfat Tris-buffered saline supplemented with Tween-20 (containing 0.1% Tween-20) at room temperature for 2 h. The membranes were incubated with the following primary antibodies: MLCK (1:1,000, Sigma, M7905), p-MLC (phosphorylated myosin light chain) (S20) (1:5,000, Abcam, ab2480), MLC (1:1,000, Santa Cruz, sc-365243/sc-293136), eNOS (1:1,000, Immunoway, YT3174), and β-actin (1:10,000, Santa Cruz, sc-47778).

### 2.8 Measurement of NO in rabbit arterial walls

The treated arterial tissue was homogenized and centrifuged, and the supernatant was collected and kept on ice for analysis. The total NO concentration in the arterial wall was measured using a commercial assay kit (Elabscience, E-BC-K035-M).

### 2.9 Noninvasive ultrasound assessment of endothelial function in the left external iliac artery

After the abdominal and left groin areas of the rabbits were shaved, FMD of the left external iliac artery was assessed using transcutaneous ultrasound. Ultrasound gel was applied to the left groin. A GE Vivid E9 (USA) ultrasound system with a 9L8 MHz probe was used to locate the bifurcation of the left common iliac artery and track the external iliac artery, obtaining longitudinal images with the beam perpendicular to the arterial wall. The baseline end-diastolic diameter (D0) of the external iliac artery was measured. A cuff was then inflated to 240 mmHg for 5 min at the left hindlimb, and the maximum end-diastolic diameter (D1) was measured after sudden deflation. The procedure was repeated three times, and the average of the three measurements was calculated for each rabbit. Measurements with a coefficient of variation >15% were excluded and repeated. FMD (%) was determined as (D1−D0)/D0 × 100% to evaluate endothelium-dependent dilation (19). The ultrasound operator was blinded to group allocations (see Section 2.12).

### 2.10 Analysis of the *ex vivo* arterial ring relaxation function

Thoracic aortic rings from rabbits were preserved with intact endothelium. Briefly, arterial rings were suspended in a 37 °C Krebs solution bath (continuously aerated with 95% O₂ and 5% CO₂), and tension was recorded using a BL-420 F system (Chengdu Taimeng Science and Technology Co., Ltd., China). After equilibration at 2 g of resting tension for 45 min, the rings were preactivated with 80 mM potassium chloride to assess functional integrity and enhance the contractile response, followed by precontraction with 1 μmol/L phenylephrine. Upon reaching stable contraction, cumulative concentrations of ACh or sodium nitroprusside (SNP; 0.001, 0.01, 0.1, 1, and 10 μmol/L) were added. Relaxation was expressed as a percentage of the precontraction amplitude.

### 2.11 Analysis

Data were analyzed using SPSS 25.0 and graphed with GraphPad Prism. WB band intensities were quantified in Fiji (ImageJ) and are expressed as target protein/internal control ratios. Categorical data are presented as *n* (%) and were compared by the chi-square test. Continuous data were first tested for normality (Shapiro–Wilk test) and homogeneity of variance (Levene's test). Data are presented as the mean ± SD for normally distributed variables or as the median (P25, P75) for nonnormally distributed variables.

Between-group comparisons: For normally distributed data, one-way ANOVA was used to compare differences among the three groups. When the overall *F* test was significant (*P* < 0.05), *post hoc* pairwise comparisons were performed using the LSD test (when the variances were equal) or Tamhane's T2 test (when the variances were unequal). For nonnormally distributed data, the Kruskal–Wallis test was used, followed by Dunn's *post hoc* test for pairwise comparisons when the overall test was significant.

Specific endpoints and their statistical tests: One-way ANOVA: PRAT weight, ox-LDL, Glu, FMD, protein expression (MLCK, p-MLC/MLC, eNOS), NO levels, and relaxation rates at each individual concentration (all ACh concentrations and all SNP concentrations except 0.1 μmol/L). Kruskal–Wallis test: Body weight, TC, TG, HDL-C, VLDL, LDL-C, and relaxation rate at 0.1 μmol/L SNP.

Two-way repeated-measures ANOVA for dose-response curves: For ACh-induced relaxation (0.001–10 μmol/L) and SNP-induced relaxation (excluding 0.1 μmol/L), a two-way repeated-measures ANOVA was performed, with Group as the between-subjects factor and Concentration as the within-subjects factor. Mauchly's test was used to assess sphericity; when violated, Greenhouse–Geisser correction was applied. Main effects (Group, Concentration) and the Group × Concentration interaction were evaluated. When a significant interaction was detected, simple effects at each concentration were further analyzed using one-way ANOVA as described above.

Correlation analyses: Given that most variables exhibited non-normal distributions, Spearman's rank correlation coefficient (ρ) was used to assess relationships among metabolic parameters and endothelium-dependent relaxation rates. For multiple comparisons (9 metabolic parameters × 5 vascular endpoints), the Bonferroni correction was applied (significance threshold: *P* < 0.00111).

### 2.12 Blinding

Group allocation was performed by an independent investigator who was not involved in the experiments. The ML-7 administrator was aware of the allocation but did not participate in the data collection or analysis. Outcome-specific blinding: FMD: A blinded technician performed ultrasound; a blinded analyst calculated FMD. Vascular rings: Blinded investigators performed all procedures using coded files; analysis was completed before decoding. WB: Blinded investigators performed the experiments; band quantification was performed by another blinded investigator using coded images. Biochemical assays: Laboratory personnel blinded to the allocations processed coded samples. A blinded statistician performed all analyses; the code was broken after final data verification. Reagent information: Detailed information regarding catalog numbers and lot numbers for all reagents and kits is available from the corresponding author upon reasonable request.

## 3 Results

### 3.1 Successful establishment of an HFD-induced obesity model

The initial body weights did not significantly differ among the groups. From week 2 onward, compared with the control group, both the HFD and ML-7 groups exhibited significantly greater body weights (*P* ≤ 0.001), with no difference between the two groups. This trend persisted until the end of the experiment. By week 8, compared with the control group, the HFD group showed a 27.85% increase in body weight (>25%), meeting the criteria for an obesity model (24).

### 3.2 PRAT deposition and dyslipidemia in obese rabbits with limited efficacy of ML-7 intervention

**Figure 1:**
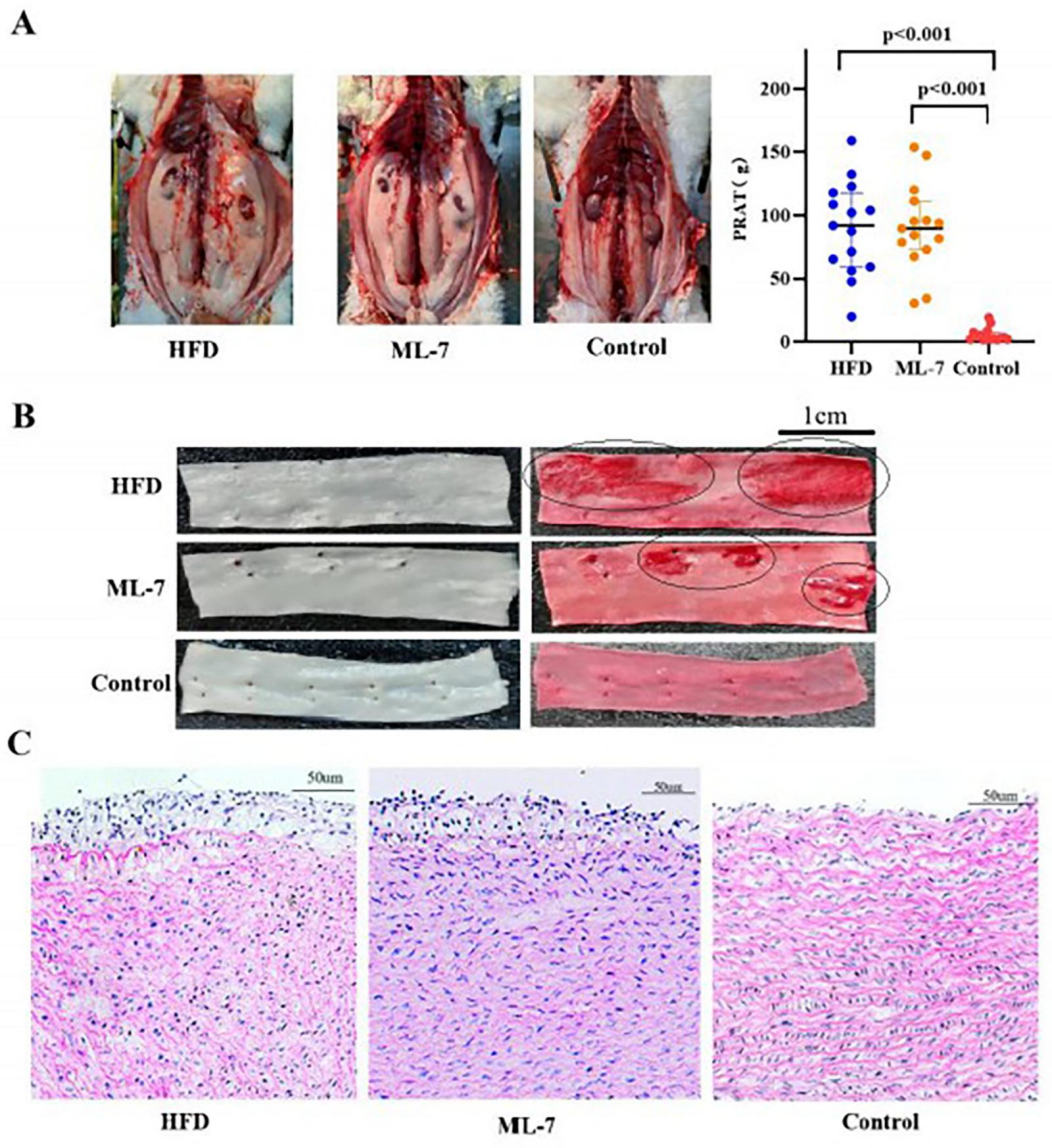
Effects of a HFD and ML-7 on PRAT accumulation and aortic morphology. **(A)** Perirenal adipose tissue (PRAT) weight in each group. **(B)** Representative Oil Red 0 staining of aortic arches showing lipid deposition (red). Scale bars = 1 cm. **(C)** Representative H&E staining of aortic sections showing structural changes. Scale bars = 50 pm. Data are presented as the mean ± SD (*n* = 15 per group for PRAT weight; *n* = 9 per group for Oil Red 0 staining; *n* = 3 per group for hematoxylin and eosin staining). Statistical analysis: one-way ANOVA with the LSD *post hoc* test. *P < 0.05 vs. control group.

Compared with the control group, both the HFD and ML-7 groups showed significant increases in PRAT, with no significant difference between the two groups (Figure 1A). After 8 weeks of feeding, the TC, LDL-C and ox-LDL levels in the HFD and ML-7 groups were significantly greater than those in the control group, with the ox-LDL levels in the ML-7 group exceeding those in the HFD group (*P* < 0.001). Glu levels were significantly elevated in the HFD group relative to those in the control group, but ML-7 administration significantly decreased these levels (Table 1). The results indicated that HFD-fed rabbits developed obesity with pronounced visceral fat deposition, dyslipidemia (increased TC, LDL-C, and ox-LDL), and hyperglycemia, which contributed to a higher risk of AS and cardiovascular disorders. However, although ML-7 alleviated hyperglycemia, it did not improve the remaining metabolic parameters.

**Table 1:** Comparison of blood lipid and glucose levels in each group.

Parameter	HFD (*n* = 10)	ML-7 (*n* = 10)	Control (*n* = 10)	H/F	*P*
TC (mmol/L)	8.0 (3.7, 21.5)*	13.2 (10.2, 17.5)*	2.2 (1.5, 3.7)	13.9	<0.001
TG (mmol/L)	57.0 (38.5, 129.0)	64.3 (34.5, 187.4)	41.2 (33.1, 70.8)	1.9	0.378
HDL-C (mmol/L)	1.6 (1.1, 3.6)	1.8 (1.0, 5.3)	1.2 (1.0, 2.0)	1.9	0.378
LDL-C (mmol/L)	7.2 (3.1, 12.9)*	13.9 (6.9, 22.6)*	1.7 (0.9, 2.8)	16.2	<0.001
VLDL (mmol/L)	1.5 (0.7, 5.4)	1.4 (0.6, 3.4)	1.1 (0.5, 3.1)	0.6	0.735
ox-LDL (mmol/L)	71.6 ± 5.7*	95.6 ± 3.2*^†^	43.1 ± 3.4	385.9	<0.001
Fasting glucose (mmol/L)	1.9 ± 0.5*	1.5 ± 0.4^†^	1.5 ± 0.3	3.4	0.047

Data are presented as the mean ± SD for normally distributed variables (ox-LDL and fasting glucose) or as the median (25th and 75th percentiles) for nonnormally distributed variables (TC, TG, HDL-C, LDL-C, and VLDL). Statistical analysis: For normally distributed data, one-way ANOVA was used; when the overall *F*-test was significant (*P* < 0.05), *post hoc* pairwise comparisons were performed using the LSD test. For non-normally distributed data, the Kruskal–Wallis test was used; when the overall test was significant, *post hoc* pairwise comparisons were performed using Dunn's test with Bonferroni correction.

TC, total cholesterol; TG, triglycerides; HDL-C, high-density lipoprotein cholesterol; LDL-C, low-density lipoprotein cholesterol; VLDL, very-low-density lipoprotein; ox-LDL, oxidized low-density lipoprotein. Normal reference ranges for New Zealand White rabbits [based on Wu et al., 2020 (25)]: TC, 0.70–2.86 mmol/L; TG, 0.37–2.05 mmol/L; LDL-C, 0.13–1.77 mmol/L; HDL-C, 0.39–0.93 mmol/L. The control group values in this study fall within these ranges. Note that reference ranges may vary by strain, age, and diet; the control group values in this study fall within these ranges.

* *P* < 0.05 vs. control group.

† *P* < 0.05 vs. HFD group.

### 3.3 Results of aortic ORO staining and HE staining in each group

At 8 weeks, visible differences in aortic wall lesions were observed among the groups. Compared with the control group, both the HFD and ML-7 groups exhibited significant ORO staining for plaques, although ML-7 intervention reduced the plaque number and area (Figure 1B). HE staining revealed significant structural damage to the aortic endothelium of obese rabbits, with marked thickening of the aortic intima accompanied by abundant foam cells, lipid deposition, and disorganized smooth muscle cells in the medial layer. Overall, these pathological changes were partially alleviated in the ML-7 group (Figure 1C).

### 3.4 *In vivo* iliac artery ultrasound reveals that ML-7 does not improve endothelium-dependent FMD in obese rabbits

**Figure 2:**
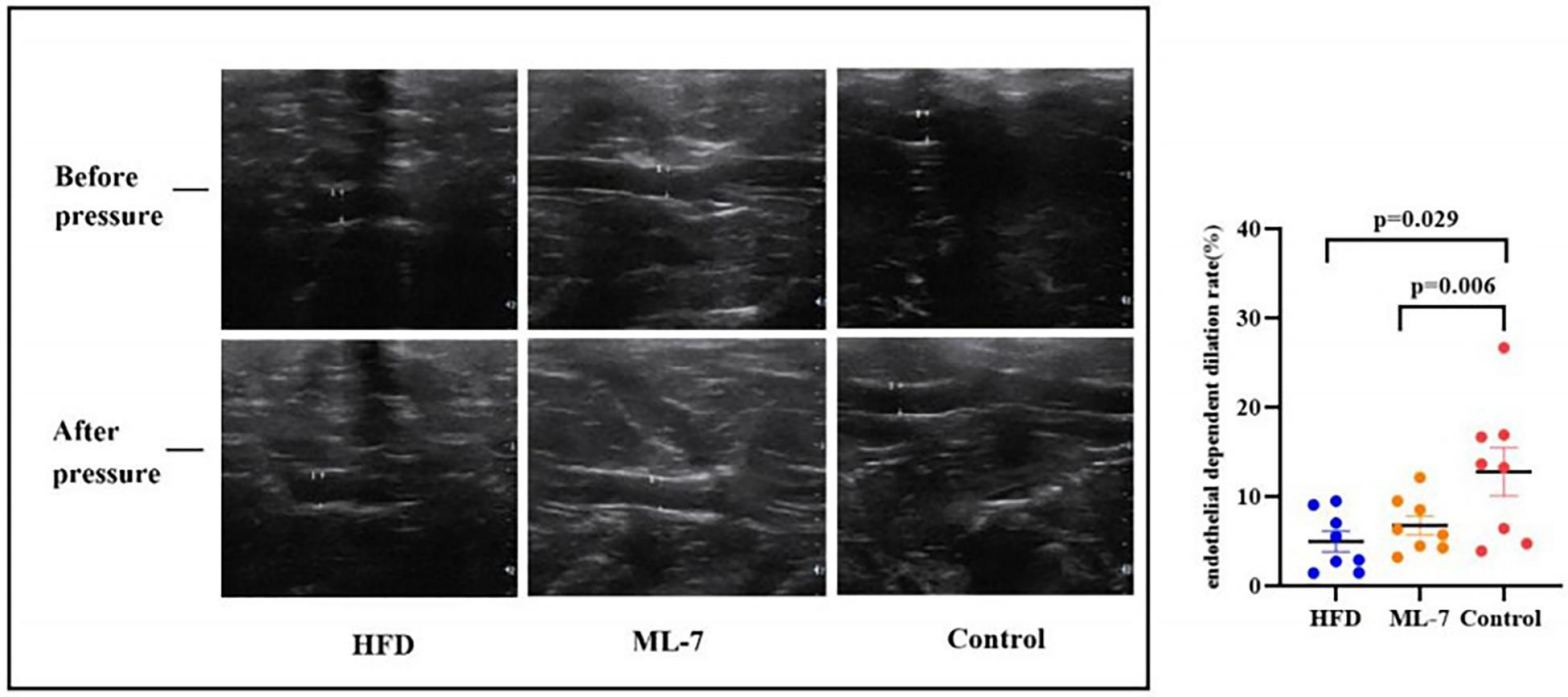
*In vivo* assessment of endothelial function by flow-mediated dilation (FMD). FMD of the left external iliac artery was measured by transcutaneous ultrasound. Endothelium- dependent dilation rate (%) = (DI—DO)/DO × 100, where DO is the baseline diameter (before pressure) and D 1 is the after-pressure diameter of the left external iliac artery. The data are presented as the mean ± SD (*n* = 8 per group). Statistical analysis: one-way ANOVA with the LSD *post hoc* test. *P < 0.05 vs. control group. Exact *P* values: *P* = 0.029, HFD vs. control group; *P* = 0.006, ML-7 group vs. control group; *P* = 0.486, ML-7 group vs. HFD group.

FMD of the external iliac artery, assessed by transcutaneous ultrasound, was used to evaluate *in vivo* endothelial function. Compared with the control group, both the HFD and ML-7 groups exhibited significantly lower FMD (*P* = 0.029/0.006), confirming obesity-induced ED *in vivo*. Critically, there was no significant difference in FMD between the ML-7 and HFD groups (*P* = 0.486; Figure 2), indicating that ML-7 treatment failed to improve endothelium-dependent dilation in the conductance arteries of living animals.

### 3.5 ML-7 ameliorates endothelium-dependent relaxation dysfunction in HFD-induced obese rabbits

**Figure 3:**
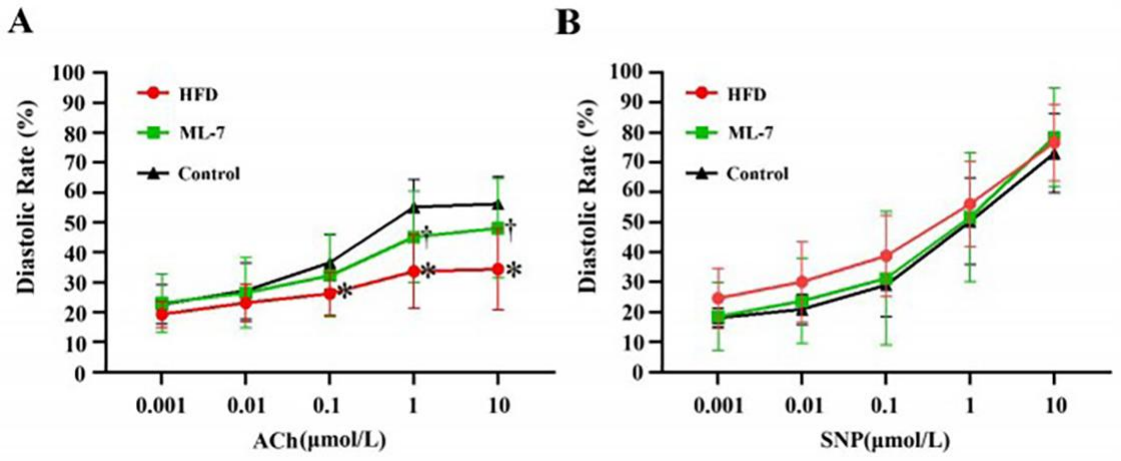
*Ex vivo* assessment of vascular relaxation function in aortic rings. **(A)** Endothelium-dependent relaxation in response to cumulative concentrations of acetylcholine (ACh). **(B)** Endothelium-independent relaxation in response to sodium nitroprusside (SNP). The data are presented as the meant SD (*n* = 7 per group). Statistical analysis: For ACh, two-way repeated-measures ANOVA with Greenhouse-Geisser correction revealed a significant Group × Concentration interaction (*p* = 0.013). Simple effects at each concentration were analyzed by one-way ANOVA followed by the Bonferroni *post hoc* correction. For SNP (excluding 0.1 ‘Amon), two-way repeated-measures ANOVA revealed no significant Group effect (*p* = 0.633) or interaction (*p* = 0.648). At 0.1 gmol/L SNP, the results of the Kruskal–Wallis test revealed no differences among the groups (*p* = 0.407). * *P* < 0.05, vs. control group,/*P* < 0.05, ML-7 group vs. HFD group.

Two-way repeated-measures ANOVA revealed a significant main effect of ACh Concentration [F(1.499, 25.477) = 101.45, *p* < 0.001, partial *η*² = 0.856] and a significant Concentration × Group interaction [F(2.997, 25.477) = 4.36, *p* = 0.013, partial *η*² = 0.339], indicating that the effect of Group differed across ACh concentrations. Simple effect analysis at each concentration (Figure 3A) revealed that compared with the control group, the HFD group exhibited significantly reduced relaxation at 0.1, 1, and 10 μmol/L ACh (all *p* < 0.05). ML-7 treatment significantly improved relaxation compared with that in the HFD group at 1 and 10 μmol/L ACh (both *p* < 0.05) but had no effect at other concentrations (all *p* > 0.05). Of note, the improvement by ML-7 was observed only at the two highest ACh concentrations (1 and 10 μmol/L) and did not restore relaxation to control levels at any concentration, indicating a partial and concentration-limited effect.

For SNP-induced relaxation, two-way repeated-measures ANOVA revealed a significant Concentration effect [F(1.414, 24.036) = 290.37, *p* < 0.001, partial *η*² = 0.945], with no significant Group effect or interaction (both *p* > 0.05) (Figure 3B). At 0.1 μmol/L SNP, the results of the Kruskal–Wallis test confirmed that there were no differences among the groups (*p* = 0.407). These results indicate that the impaired relaxation in HFD-induced obese rabbits is endothelium dependent and that ML-7 partially ameliorates this dysfunction without affecting smooth muscle responsiveness to NO.

### 3.6 Correlation analysis of *ex vivo* arterial ring relaxation rates with other indicators

Spearman correlation analysis revealed that PRAT was positively correlated with body weight (*ρ* = 0.807, *P* < 0.001) and negatively correlated with endothelium-dependent relaxation rates at multiple ACh concentrations. After applying a Bonferroni correction for 45 tests (adjusted significance threshold of *P* < 0.00111), PRAT remained significantly correlated with relaxation rates at an ACh concentration of 1 μmol/L (*ρ* = −0.648, *P* = 0.001) and an ACh concentration of 10 μmol/L (*ρ* = −0.691, *P* = 0.001), whereas the correlation at an ACh concentration of 0.1 μmol/L (*ρ* = −0.591, *P* = 0.005) was no longer significant. TC, LDL-C, and ox-LDL levels were significantly positively correlated with body weight and PRAT (*ρ* ranged from 0.586–0.622, all *P* < 0.001), all of which remained significant after correction. No significant correlations were detected between any metabolic parameters and relaxation rates at any SNP concentration (all *P* > 0.05). Detailed correlation coefficients and *P* values are presented in Table 2.

**Table 2:** Correlation analysis of the diastolic rate of isolated arterial rings with blood lipids, glucose, body weight and PRAT.

Variables				Diastolic rate				
		Weight	Perirenal fat	Ach0.001	Ach0.01	Ach0.1	Ach1	Ach10
Weight	*ρ*	1.000	.807	−0.076	−0.207	−0.343	−0.282	−0.290
	*P*	.	0^†^	0.745	0.368	0.128	0.216	0.203
Perirenal fat	*ρ*	.807	1.000	−0.349	−0.403	−.591	−.648	−.691
	*P*	0	.	0.121	0.070	0.005	0.001^†^	0.001^†^
Glu	*ρ*	0.147	0.065	−0.207	−0.256	−0.255	−0.169	−0.178
	*P*	0.438	0.735	0.395	0.289	0.293	0.490	0.465
TC	*ρ*	.606	.586	−0.001	−0.036	−0.269	−0.414	−0.362
	*P*	0^†^	0.001^†^	0.997	0.887	0.280	0.088	0.140
TG	*ρ*	0.181	0.253	0.080	0.039	−0.018	−0.164	−0.212
	*P*	0.340	0.178	0.745	0.872	0.943	0.502	0.383
HDL-C	*ρ*	0.181	0.253	0.080	0.039	−0.018	−0.164	−0.212
	*P*	0.340	0.178	0.745	0.872	0.943	0.502	0.383
LDL-C	*ρ*	.602	.586	−0.035	−0.068	−0.261	−0.409	−0.377
	*P*	0^†^	0.001^†^	0.887	0.781	0.280	0.082	0.111
ox-LDL-C	*ρ*	.622	.609	0.140	0.133	−0.084	−0.247	−0.247
	*P*	0^†^	0^†^	0.567	0.586	0.732	0.307	0.307
VLDL	*ρ*	0.011	0.031	−0.298	−0.319	−0.362	−0.422	−0.401
	*P*	0.956	0.875	0.229	0.197	0.140	0.081	0.099

PRAT, perirenal adipose tissue; TC, total cholesterol; TG, triglycerides; HDL-C, high-density lipoprotein cholesterol; LDL-C, low-density lipoprotein cholesterol; VLDL, very-low-density lipoprotein; ox-LDL-C, oxidized low-density lipoprotein cholesterol; ox-LDL, oxidized low-density lipoprotein; Glu, fasting glucose. Bivariate correlation analysis was used to assess the correlation of risk factors with endothelium-dependent vasodilation.

† *P* < 0.00111 after Bonferroni correction for 45 tests.

### 3.7 HFD-induced obese rabbits exhibited significantly increased arterial wall MLCK expression and MLC phosphorylation levels, which were reversed by ML-7 intervention

**Figure 4:**
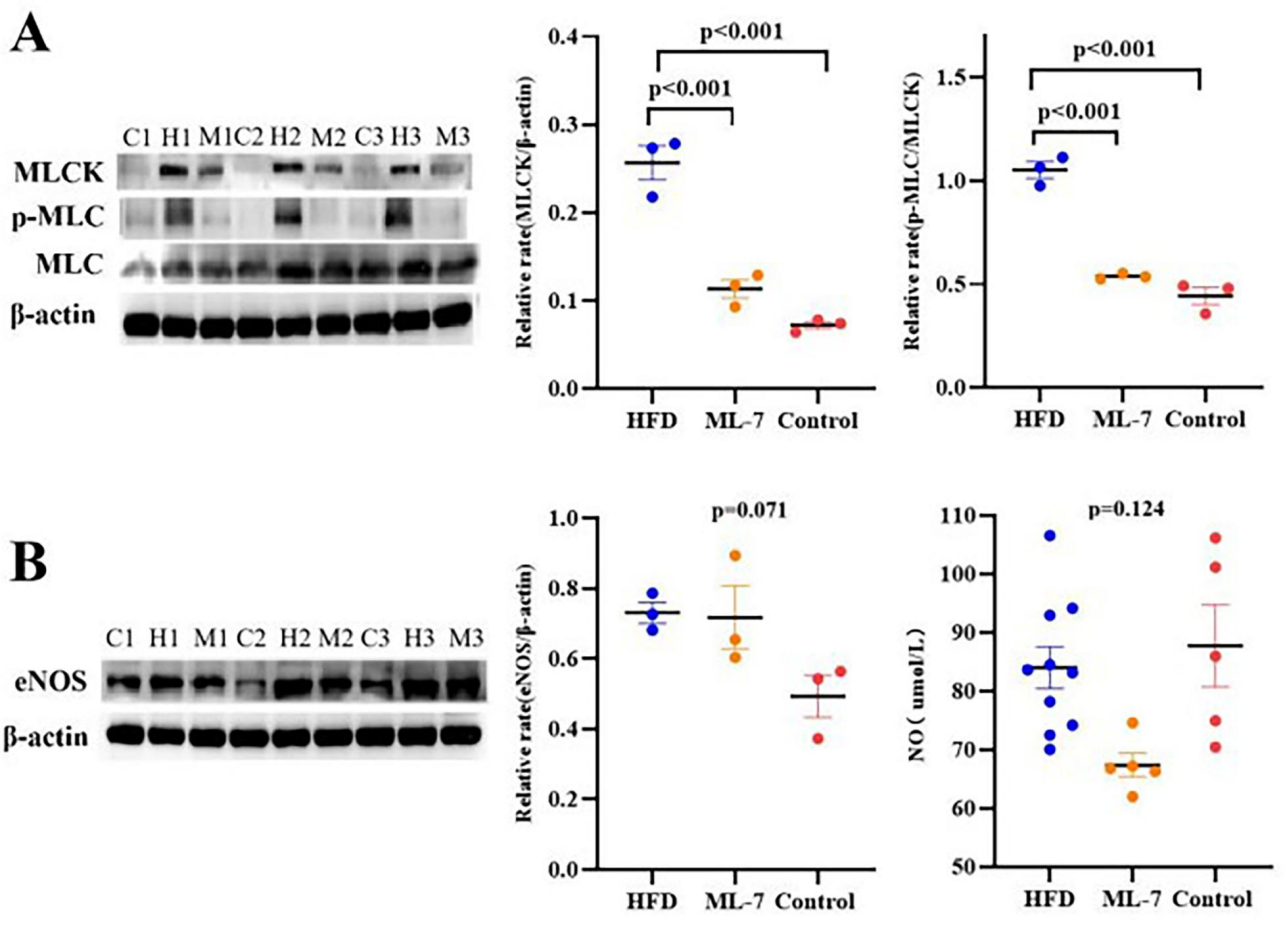
Protein expression and nitric oxide (NO) levels in aortic tissue. **(A)** Representative Western blots and quantitative analysis of MLCK (myosin light chain kinase), p-MLC (phosphorylated myosin light chain), and total MLC (myosin light chain). **(B)** eNOS (endothelial nitric oxide synthase) protein expression and NO levels. (3-actin was used as a loading control. The data are presented as the mean ± SD (*n* = 3 per group for Western blot; *n* = 5 per group for NO measurement). Statistical analysis: one-way ANOVA with the LSD *post hoc* test. No significant differences in eNOS expression (*P* = 0.071) or NO levels (*P* = 0.124) were detected among the groups.

WB analysis revealed that compared with the control and ML-7 groups, the HFD group had significantly higher aortic MLCK expression and p-MLC/MLC ratios (*P* < 0.001), with no significant differences among the other groups (all *P* > 0.05) (Figure 4A).

### 3.8 No significant differences in aortic eNOS expression or NO levels among groups

WB analysis revealed that aortic eNOS expression was comparable among the three groups (*P* = 0.071/0.124). Similarly, the NO levels in the arterial walls did not differ significantly between the groups (Figure 4B). These findings indicate that the obesity-induced ED observed in this study occurred independently of changes in eNOS protein abundance or total NO content.

## 4 Discussion

Obesity can independently promote the development of CVD and increase CVD-related mortality, distinct from other risk factors (7). Although the precise pathophysiology remains incompletely elucidated, our study investigated obesity-induced vascular dysfunction by focusing on its earliest manifestation—ED. Our findings demonstrated that obesity triggers structural and functional vascular impairment in rabbits, characterized by arterial endothelial damage and compromised endothelium-dependent vasorelaxation. Mechanistically, this obesity-associated ED is correlated with a multifactorial interplay of dyslipidemia, oxidative stress, visceral adiposity, and upregulation of the MLCK pathway, with a consequent increase in MLC phosphorylation levels. However, the therapeutic effect of MLCK inhibition differed markedly between the *in vivo* and *ex vivo* assessments, as discussed below.

LDL and its oxidized form (ox-LDL) are well-established pathogenic drivers of atherosclerosis, where LDL-C serves as the classical biomarker of LDL (26). Consistent with this paradigm, our study demonstrated that obese rabbits exhibited significantly elevated levels of both LDL-C and ox-LDL. The excessive retention of ox-LDL in the subendothelial space and its subsequent uptake by macrophages lead to foam cell formation, promoting ED. Concurrently, ED facilitates the transport of LDL to the subendothelial space, where it is further oxidized into ox-LDL, creating a vicious cycle. Notably, the marked increase in ox-LDL levels observed in our model not only reflects enhanced LDL oxidation but also implies a state of systemic oxidative stress. Multiple enzymatic pathways contribute to this process, including vascular nicotinamide adenine dinucleotide phosphate oxidase, myeloperoxidase activity, mitochondrial dysfunction, and eNOS uncoupling [as reported in other studies (27, 28)], all of which generate reactive oxygen species that oxidize LDL. Importantly, a HFD not only induces hyperlipidemia but also enhances oxidative stress and inflammation, perpetuating a vicious cycle (29), which aligns with our findings.

A notable finding of this study is that despite clear impairment of endothelium-dependent relaxation in HFD-fed rabbits, aortic eNOS protein expression and total NO content remained unchanged. This indicates that in our rabbit model of diet-induced obesity, endothelial dysfunction is not driven by reduced eNOS abundance or gross NO deficiency. Instead, it may result from altered signal transduction from the endothelium to the smooth muscle, or from increased smooth muscle contractility downstream of NO. This interpretation is supported by the observation that ML-7, an inhibitor of smooth muscle MLCK, partially rescued relaxation without affecting eNOS or NO levels. Thus, our data suggest that obesity-induced impairment of endothelium-dependent relaxation can manifest even when the eNOS/NO pathway is intact, and that targeting smooth muscle contractility may offer a mechanistic explanation for the partial functional rescue.

Visceral and ectopic fat are key drivers of adverse cardiometabolic outcomes in patients with obesity, with visceral fat closely linked to arterial function (30). PRAT, a major component of visceral fat, secretes adipokines and proinflammatory cytokines, and its thickness is positively correlated with the visceral fat area (31, 32). Recent studies suggest that PRAT plays a significant role in maintaining cardiovascular and renal homeostasis (23). Our experimental findings demonstrated that diet-induced obese rabbits developed significant PRAT accumulation. Importantly, PRAT correlated positively with weight but negatively with aortic relaxation at all the tested ACh concentrations (0.1, 1, and 10 μmol/L; all *P* < 0.05). Furthermore, serum lipid parameters, including TC, LDL-C, and ox-LDL, were positively correlated with both weight and PRAT volume. These findings collectively suggested a pathogenic interplay between adiposity, dyslipidemia, and vascular dysfunction, with PRAT accumulation showing particularly strong associations with impaired endothelium-dependent vasorelaxation. Emerging evidence suggests that PRAT may influence cardiovascular function through multiple pathways, including (1) neural reflex modulation, (2) adipokine secretion, and (3) fat–kidney crosstalk (31). Mechanistically, PRAT expansion may promote ED via increased tetrahydrobiopterin oxidation and subsequent superoxide overproduction, ultimately reducing NO bioavailability (33). However, the exact pathogenic mechanisms require further elucidation.

This study demonstrated that ED in obese rabbits is associated with MLCK overexpression and increased MLC phosphorylation, dyslipidemia, and perirenal fat deposition, whereas ML-7 intervention improves endothelium-dependent relaxation. Notably, compared with control/HFD-treated rabbits, ML-7-treated rabbits demonstrated paradoxical increases in ox-LDL levels (*P* < 0.001), which were accompanied by a trend toward decreased NO content that did not reach statistical significance, suggesting a potential ML-7-induced oxidative stress response that warrants further investigation.

An unexpected finding was the significant increase in circulating ox-LDL levels in ML-7-treated rabbits compared to the HFD group, despite improvements in *ex vivo* relaxation. The mechanism underlying this paradoxical observation is unclear. We did not directly measure vascular reactive oxygen species production, eNOS uncoupling, or antioxidant enzyme activities. Therefore, any interpretation involving oxidative stress remains speculative. It is possible that ML-7 has off-target effects on lipid metabolism or LDL oxidation that are independent of its MLCK inhibitory action. Alternatively, the increase in ox-LDL may reflect altered clearance or distribution rather than enhanced oxidation. Future studies incorporating direct measurements of arterial reactive oxygen species (e.g., dihydroethidium staining), eNOS dimer/monomer ratio, and plasma antioxidant capacity are necessary to resolve this paradox.

A key finding of this study was the discrepancy between *in vivo* and *ex vivo* assessments of endothelial function: although ML-7 partially improved ACh-induced relaxation in isolated aortic rings, it failed to improve FMD in the iliac artery of the same animals (Figure 2). This dissociation has important implications. First, as a noninvasive technique, FMD may lack the sensitivity to detect subtle, partial improvements in endothelial function, particularly in conductance vessels where the response might be diluted by systemic neurohormonal influences. Second, FMD measurements were performed in conscious, mildly restrained rabbits, a condition that may have induced stress, anxiety, and subsequent sympathetic activation. These physiological responses can influence vascular tone and endothelial function, potentially masking modest treatment effects that are more readily detectable in isolated arterial rings under controlled *ex vivo* conditions. Third, atherosclerosis is a systemic disease, but plaque characteristics and functional alterations can vary significantly across different vascular beds (34). The therapeutic effect of ML-7 might be more pronounced in the thoracic aorta than in the peripheral iliac artery. Finally, the paradoxical increase in oxidative stress markers (e.g., ox-LDL) observed in the ML-7 group may have counteracted any potential benefits on FMD, which is a response largely dependent on acute NO bioavailability. Regardless of the explanation, the lack of FMD improvement indicates that the *ex vivo* benefit of ML-7 does not translate to improved *in vivo* endothelial function in conductance arteries under our experimental conditions. This finding tempers the translational significance of MLCK inhibition for obesity-related vascular dysfunction, at least at the dose and duration tested.

In summary, this study demonstrates that HFD-induced obesity in rabbits impairs endothelium-dependent relaxation, which is associated with dyslipidemia, visceral fat accumulation, and upregulation of the MLCK/p-MLC pathway. The eNOS/NO pathway remains unchanged. The MLCK inhibitor ML-7 partially improves *ex vivo* ACh-induced relaxation in a concentration-dependent manner, an effect likely mediated by reduced smooth muscle contractility rather than restoration of NO bioavailability. However, this effect does not translate into improved FMD *in vivo*. Moreover, ML-7 treatment unexpectedly increased circulating ox-LDL levels, the mechanism of which remains unknown. These findings indicate that MLCK upregulation is a correlate of obesity-induced vascular dysfunction, but its inhibition yields only partial, *ex vivo*-limited benefits in this model. The therapeutic potential of MLCK inhibitors for obesity-related vascular disease remains to be established, and future studies should directly assess vascular reactive oxygen species, eNOS coupling, and explore alternative dosing or delivery strategies to achieve *in vivo* efficacy.

## 5 Limitations

Several limitations of this study should be acknowledged. First, we did not directly measure reactive oxygen species production or eNOS coupling status; therefore, any interpretation regarding oxidative stress as an explanation for the increased ox-LDL levels remains speculative. Second, no formal *a priori* sample size calculation was performed; future studies should incorporate sample size estimation based on the effect sizes reported here. Third, our study was conducted in a rabbit model of diet-induced obesity, so extrapolation to human pathophysiology should be made with caution. Fourth, although ML-7 is a selective MLCK inhibitor, we cannot completely rule out off-target effects that may have contributed to the observed changes. Fifth, the dissociation between *ex vivo* and *in vivo* findings (ACh-induced relaxation vs. FMD) indicates that the partial benefit of ML-7 does not translate to improved endothelial function in conductance arteries in living animals, which limits the physiological and translational significance of the *ex vivo* results.

## Data Availability

The raw data supporting the conclusions of this article will be made available by the authors, without undue reservation.
